# An extremely rare serovar of *Salmonella enterica* (Yopougon) discovered in a Western Whip Snake (*Hierophis viridiflavus*) from Montecristo Island, Italy: case report and review

**DOI:** 10.1007/s00203-023-03772-w

**Published:** 2024-01-03

**Authors:** Andrea Francesco De Bene, Valeria Russini, Carlo Corradini, Silvia Vita, Sabrina Pecchi, Maria Laura De Marchis, Giuliana Terracciano, Claudia Focardi, Alessandro Montemaggiori, Marco Alberto Luca Zuffi, François-Xavier Weill, Teresa Bossù

**Affiliations:** 1 Istituto Zooprofilattico Sperimentale del Lazio e della Toscana “M. Aleandri”, Sezione di Roma, 00178 Rome, Italy; 2 Istituto Zooprofilattico Sperimentale del Lazio e della Toscana “M. Aleandri”, UOT Toscana Nord, 56123 Pisa, Italy; 3 Istituto Zooprofilattico Sperimentale del Lazio e della Toscana “M. Aleandri”, UOT Toscana Centro, 50010 San Martino Alla Palma, FI Italy; 4 Dipartimento di Biologia e Biotecnologie “Charles Darwin”, Università degli Studi di Roma “La Sapienza”, 00185 Rome, Italy; 5 Museo Di Storia Naturale, Università Di Pisa, 56011 Calci, PI Italy; 6 Institut Pasteur, Unité Des Bactéries Pathogènes Entériques, Centre Collaborateur de L’Organisation Mondiale de La Santé Pour Les Salmonella, Université Paris Cité, 75015 Paris, France

**Keywords:** Isles microbiology, Pathogens’ migrations, Rare Salmonella, Wild snakes

## Abstract

Reptiles, including snakes, can be asymptomatically infected with multiple pathogen microorganisms, including *Salmonella* spp., which is considered an important concern for public and animal health. Small and uninhabited isles are quite ecologically different from mainland and represent interesting fields of study, to discover unexpected biological and microbiological aspects of their wild inhabitants. This work reports the presence of the very rare *Salmonella enterica* serovar Yopougon, isolated in a carcass of a native wild snake (*Hierophis viridiflavus*) from an Italian uninhabited island of Mediterranean Sea, Montecristo. To our knowledge, *S. enterica* serovar Yopougon was previously isolated only once 34 years earlier in Ivory Coast, from a human fecal sample. In the present study, we present the genomic characterization of the new isolate, the phylogenetic comparison with the previously isolated *S. enterica* serovar Yopougon strain of human origin and with other sequences available in public databases. In addition, an extensive review of available data in the literature and from our case history is provided. Our finding represents an example of the ability of some pathogens to travel for very long distances within their hosts and then to infect others, even from different taxa.

**Supplementary Information:**

The online version contains supplementary material available at 10.1007/s00203-023-03772-w.

## Introduction

Reptiles can host asymptomatically pathogenic microorganisms and serve as potential reservoirs of infection for humans, pets, and other reptiles*.* Knowledge on the intestinal microbiota of free-living reptiles is still limited, although a greater number of studies have been conducted on some pathogens of zoonotic interest or frequently isolated in reptiles kept as pets or of commercial interest (Schmidt et al. 2014; McWhorter et al. 2021).

*Salmonella* spp. is considered a frequent commensal of the intestinal microbial flora of most snakes and reptiles in general, as well as many other animal species, which intermittently spread the bacterium in the environment through their feces (Briones et al. 2004; Köbölkuti et al. 2009; McWhorter et al. 2021). The biological role of free-living snakes in the transmission of bacteria and parasites is still hardly explored. Due to direct contact with the reptiles themselves or environmental contamination, the consequences for public health are not negligible (Zając et al. 2016). The infection rates of *Salmonella* spp. in reptiles vary according to the geographical area considered, the host species, and among populations of captive and free-living reptiles. Moreover, fluctuations in infection rates can also be influenced by temporal or seasonal variations*.* Reptiles are generally infected with *Salmonella* through contact with food, water, and the environment (McWhorter et al. 2021).

Bacteria of the genus *Salmonella* are divided into two main species: *S. enterica* and *S. bongori*. *S. enterica* includes six subspecies: *S. enterica* subsp*. enterica* (I), *S. enterica* subsp*. salamae* (II), *S. enterica* subsp*. arizonae* (IIIa), *S. enterica* subsp*. diarizonae* (IIIb), *S. enterica* subsp*. indica* (VI), and *S. enterica* subsp*. houtenae* (IV) (Briones et al. 2004; Grimont and Weill 2007). The subspecies *S. enterica* subsp*. enterica* (I) includes approximately 2600 known serovars, characterized by a wide variety of phenotypes, genotypes, and ecological behaviors. Members of the subspecies *S. enterica* subsp*. enterica* (I) are primarily responsible for disease in humans (about 99% of all cases of salmonellosis), other mammals, and birds, whereas all other subspecies of *S. enterica* are sporadically linked to disease episodes in these animal species (Lamas et al. 2018; Pulford et al. 2019; Vila Nova et al. 2019).

The islands of the Mediterranean Sea represent excellent areas for the study of ecological phenomena related to the spread of insular diseases, because of their well-known history and the different animal populations identified so far. Montecristo Island, located in the Tyrrhenian Sea, constitutes a particularly noteworthy reality. Despite its small surface area (10.39 km^2^), it hosts two species of snakes that are particularly widespread throughout the Italian peninsula: the Western Whip Snake, *Hierophis viridiflavus* (Lacépède 1789), and the asp viper, *Vipera aspis* (Linnaeus 1758) (Vanni and Zuffi 2011; Luiselli et al. 2015).

The Western Whip Snake is a medium-sized nonvenomous colubrid (100–140 cm in total length) present in a large variety of habitats throughout Europe, especially in the (Fornasiero et al. 2007). The specimen present in Montecristo Island is approximately 30% smaller than the continental *H. viridiflavus* populations, probably due to the ecological phenomenon known as insular dwarfism (Zuffi et al. 2003; Luiselli et al. 2015). The snake is present in every type of habitat and soil found on Montecristo. The diet of this population of snakes consists of lizards, passerine birds, and secondly insects, since small mammals such as rodents, which are important components of the diet of continental conspecifics, are absent on the island territory (Luiselli et al. 2015).

## Materials and methods

### Microbiological methods for identification and serotyping

*Salmonella* spp. was isolated according to the method described in “OIE Manual for terrestrial animals 2018” (Chapter 3.9.8 par A, B, 2016; Chapter 3.3.11 A, B, 2018) and identified through cultural test. Further investigations on the strain (SAL_117300), such as serotyping analysis, according to the “ISO/TR 6579–3: 2014” standards, and molecular analysis through HTS (high throughput screening), were carried out.

### Whole genome sequencing

Genomic DNA was extracted with automatic extraction system, QIAsymphony (Qiagen, Hilden, Germany). Libraries were prepared using Nextera XT DNA Library Prep and pair-end (2 × 300 bp) run with a MiSeq sequencer (Illumina, CA, USA). Raw reads were archived in the GenBank database (NCBI-SRA) under the BioProject PRJNA998331, BioSample SAMN36701244.

The quality of raw reads was assessed with Fast QC (v0.11.5) (Andrews 2023), and low-quality reads and adapters were trimmed using Trimmomatic (v0.39) (Bolger et al. 2014) before any analysis was done using the following quality filter: minimum quality of Q30, a window size of 10 with Q20 as the average quality, and a minimum length read of 50 bp. The high-quality reads were de novo assembled into contigs using SPAdes (v3.13.0) (Bankevich et al. 2012) with the careful option on, draft assemblies were improved using Pilon (v1.23) (Walker et al. 2014), and contigs shorter than 500 bp were removed (Bushnell 2023). Plasmids were found with plasmidSPAdes (Bankevich et al. 2012). The assembly quality was assessed with QUAST (v5.0.2) (Gurevich et al. 2013). The antigenic formula was deduced *in* *silico* using SeqSero2 (v1.1.0) (Zhang et al. 2015, 2019) on the ARIES Galaxy server (Knijn et al. 2020). *In-silico* subtyping, or multi-locus sequence typing (MLST), was performed on EnteroBase platform (Zhou et al. 2020) that used the classic scheme for *Salmonella* spp. of seven housekeeping genes (*aroC*, *dnaN*, *hemD*, *hisD*, *purE*, *sucA*, *thrA*) described by Kidgell et al. (2002). On the same platform, the core genome MLST (cgMLST) analysis, that analyzed 3002 core genes of *Salmonella,* was performed. Subsequently, a comparison was performed with the most similar sample in cgMLST collected in the *Salmonella* international database based on EnteroBase (Achtman et al. 2021) that collect more than 410,000 isolates genome. The distances were visualized with GrapeTree (Zhou et al. 2018) using the algorithm RapidNJ showing the assigned hierarchical clusters (HCs), and MSTree V2, for cgMLST allelic distances. The reference strain of *S*. *enterica* serovar Yopougon (IP 7148/89) was included in the analysis, whose genome sequence is present in the *Salmonella* database on EnteroBase (Achtman et al. 2021). This reference strain was isolated from human in Ivory Coast in 1989. The identification of antimicrobial resistance genes was assessed from the assemblies using ABRicate (Seemann 2020), based on ResFinder database (Zankari et al. 2012) and performed on the ARIES Galaxy Server (Knijn et al. 2020). The presence of virulence genes was assessed using the tool VirulenceFinder v2.0.3 (Joensen et al. 2014) against the Virulence Factors of Pathogenic Bacteria (VFDB) database of core virulence genes (Liu et al. 2019). The assembled genome was annotated using RAST tool kit (RASTtk) (Brettin et al. 2015) on BV-BRC platform (Olson et al. 2023).

### Analytical methods for toxicological determinations

The following analytical methods have been used for determination of major toxicants: Strychnine, Crimidine, Metaldehyde, and Pesticides using gas chromatography–mass spectrometry (GC/MS) system by Thermo Scientific and Anticoagulant rodenticides using high-performance liquid chromatography (HPLC) system by Sciex, coupled with both fluorometric and diode array detector. QuEChERS methodology was used for the extraction and purification steps (protocol developed in house, available on request).

## Results

In October 2021, two carcasses of Western Whip Snake (*H. viridiflavus*), one male and one female, were dispatched to the Operating Territorial Unit of Pisa of the Istituto Zooprofilattico Sperimentale del Lazio e della Toscana “M. Aleandri” (IZSLT). The reptiles were found dead in Montecristo Island, a protected nature reserve located in the Tyrrhenian Sea and included in the Tuscan Archipelago National Park (42 20′05″ N, 10 18′44″ E) (Luiselli et al. 2015). The two snakes were found days earlier by agents of the national police (Carabinieri Forestali). The carcasses were then carried to the Museum of Natural History of the University of Pisa, which preserved the specimens until they were sent to the IZSLT for further diagnostic investigation.

Anatomical-pathological examination showed the death of the two snakes dated back to some days before, considering the state of dehydration and the level of exfoliation of the skin. However, no macroscopic lesions in the internal organs were observed in either animal. Furthermore, toxicological screening for metaldehyde, pesticides (carbamates, organochlorines, organophosphorus), and strychnine gave negative results. After necropsy, microbiological research was carried out on the organs of the two snakes (intestine, myocardium and a pool of organs). Microbiological investigations on female Western Whip Snake organs tested negative, whereas the male specimen intestine and myocardium tested positive for *Salmonella* spp. and *Bacillus* spp., respectively. The *Salmonella* strain (SAL_117300) was serotyped as *S. enterica* subsp*. enterica* serovar Yopougon with antigenic formula 45:z:e,n,z_15_ (hereafter referred to as *S.* Yopougon).

### Genomic analyses

**Fig. 1:**
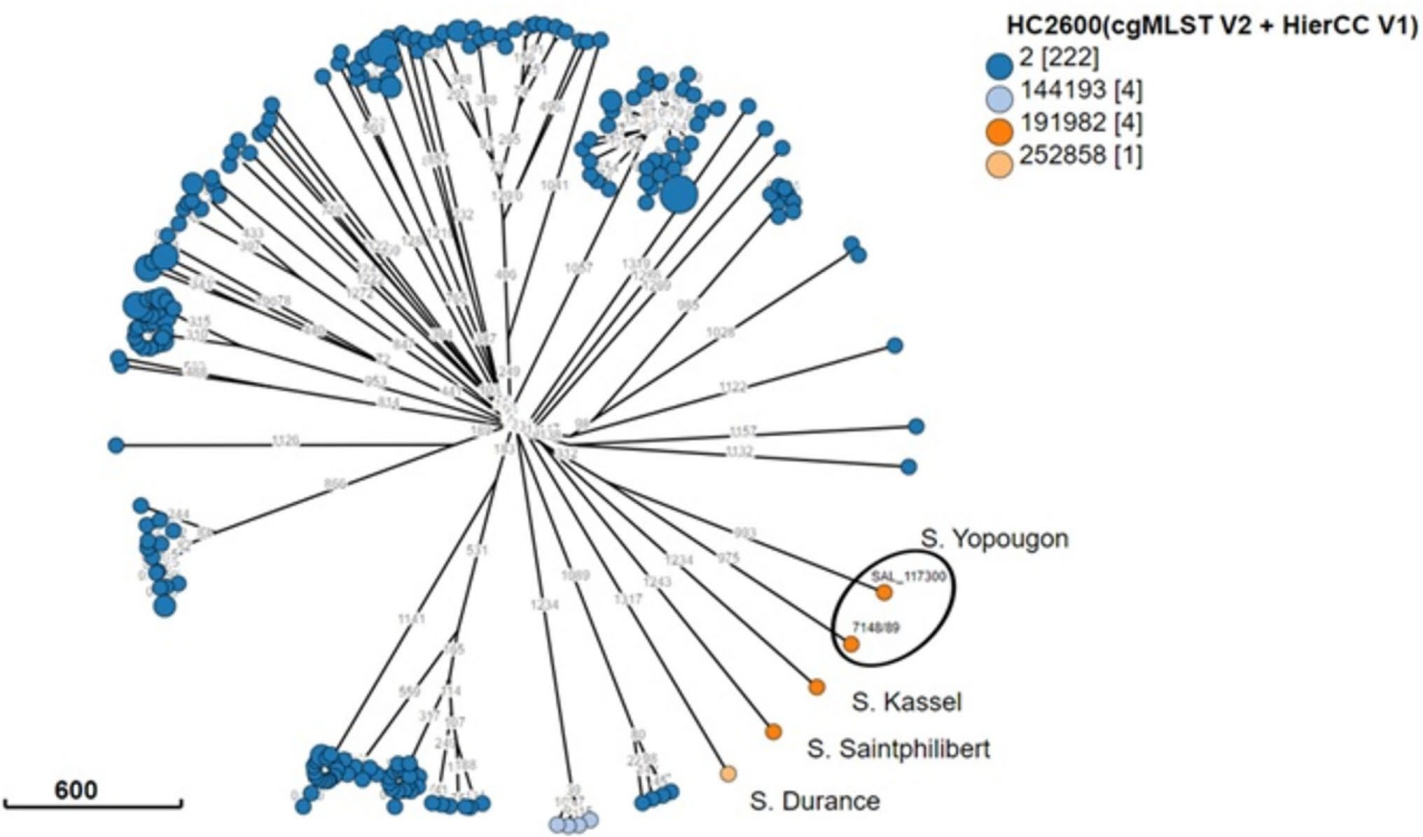
Hierarchical clusters HC2600 of the closest genomes found in EnteroBase of *S*. Yopougon SAL_117300, calculated with cgMLST. Orange—the strains belonging to the HC2600_191982, including the two *S*. Yopougon (black circle)

*In* *silico* serotyping confirmed the identification of *S.* Yopougon (O:45, *fliC*:z, and *fljB*:e,n,z_15_). The strain carries the gene *fosA7* that encodes resistance to the antibiotic fosfomycin. A new MLST, ST10543, was assigned to the strain on EnteroBase and resulted the only one belonging to this ST. We then used cgMLST, a more discriminative approach based on 3002 genes, and implemented it into EnteroBase. The used cgMLST scheme also assigned bacterial genomes to single-linkage hierarchical clusters (HCs) in real-time, at various levels of resolution, ranging from HC0 (high-resolution clusters consisting of identical genomes with no allelic differences) to HC2850 (low-resolution clusters consisting of genomes with up to 2850 allelic differences). The SAL_117300 genome was found to belong to HC2850_2 (as for subspecies *enterica* strains), HC2600_191982, and HC2000_319874. This latter group contained only two genomes: SAL_117300 and IP 7148/89, the reference strain of *S.* Yopougon. However, the allelic differences between the two genomes were quite high (*n* = 1968). Figure 1 shows the HC 2600 of the closest genomes found in EnteroBase. The closest genomes were from the following rare serovars Saintphilibert with 2571 allelic differences, Durance with 2600, Kassel with 2635, and Koblenz and Chicago with 2644 allelic differences. All other serovars were at least 2658 alleles apart and represented in a Minimum Spanning Tree shown in Figure S1.

Virulence genes found in the investigated strain are shown in Table S1. Approximately 60% of the core virulence genes was found in the genome. The annotation of the entire genome included 1,012 hypothetical proteins and 4,554 proteins with functional assignments and a genome size of 5.3 Mbp. The representation of genes found in the assembled genome is reported in Figure S2.

The only other sequenced strain of *S.* Yopougon (IP 7148/89) available was most similar to the one isolated in the snake (SAL_117300), compared to others in the public database. The IP 7148/89 strain had a genome size of 4.7 Mbp and harbored 650 hypothetical proteins and 4,116 proteins with functional assignments.

## Discussion

*S.* Yopougon is an extremely rare serovar of *S. enterica* subsp*. enterica*, and no publications or data appear in the literature. In the international database EnteroBase (Achtman et al. 2021), only one strain was referable to this serovar: *S.* Yopougon strain IP 7148/89. This strain was isolated in Ivory Coast in 1989 (ndr: plausibly in the municipal of Yopougon, in the District of Abidjan) from a sample of human feces and sequenced at the Institut Pasteur, Paris, France (FXW). The genomic analysis confirmed a closer relationship between the two Yopougon strains compared with the other serovars in public databases, but with a high allelic distance according to the long temporal range. The virulence and resistance genes found were similar in both strains, but the genome size and the annotated genes were higher in the isolate from the snake.

From a bibliographic comparison of various studies on serotyping of *Salmonella* spp., conducted in the District of Abidjan, no isolation of this serovar has ever emerged in the different matrices of human and animal origin (poultry, cattle) (Boni-Cissé et al. 2012; Bonny et al. 2015; Yao et al. 2017).

To date, there are no prevalence studies on *Salmonella* spp. or other bacterial species in free-living snakes in Italy. Few reviews relating to European snakes are available, none of which, however, included *H. viridiflavus* among the sampled species.

According to a study conducted in Poland, 45 native free-living grass snakes (*Natrix natrix*) were captured and kept under controlled laboratory conditions. It emerged that 22% (*n* = 10) were positive for *Salmonella* spp. on fecal sampling through cloacal swabs. *Salmonella* spp. was the fourth most frequently isolated pathogen in the study, after *Aeromonas hydrophila* (37.8%), *Morganella morganii* (26.7%), and *Proteus vulgaris* (24.4%). To date, the study of Pawlak and colleagues reported the highest number of free-living snakes analyzed for *Salmonella* spp. in Europe, (*n* = 45) (Pawlak et al. 2020).

Another study conducted in Poland on 16 autochthonous snakes, 15 grass snakes (*Natrix natrix*) and a smooth snake (*Coronella austriaca*), found positivity to *Salmonella* spp. in 87.5% of specimens (*n* = 14). The molecular investigations (PCR multiplex), conducted on a total of 33 *Salmonella* isolates, identified 11 distinct serovars, 9 of which belong to *S. enterica* subsp. *diarizonae* (IIIb) and 2 to *S. enterica* subsp*. enterica* (I). Positive reptiles were infected with one to four different *Salmonella* serovars at the same time (Zając et al. 2016).

A research carried out in Romania showed 62.5% of positivity for *Salmonella* spp. on autochthonous vipers (*Vipera berus*; *n* = 19). The most isolated subspecies through biochemical investigations was *S. enterica* subsp*. arizonae* (IIIa) among positive samples (Köbölkuti et al. 2009).

A noteworthy German study was conducted on free-living vipers (*Vipera berus*; *n* = 23) and grass snakes (*Natrix natrix*; *n* = 12) on the island of Hiddensee (Germany), testing swabs from pharynx and cloaca. This research demonstrated the presence of a single subspecies of *Salmonella, S. enterica* subsp*. diarizonae* (IIIb), exclusively in eight vipers (34.8%). Other potentially pathogenic bacteria isolated from swabs were *P. vulgaris*, *A. hydrophila*, *Klebsiella pneumoniae,* and *Escherichia coli* (Schmidt et al. 2014). A previous research on native snakes of the same island had identified a much lower prevalence of *Salmonella* spp. (1 positive specimens out of 22 sampled) (Krautwald-Junghanns et al. 2013), but the reason for such markedly different results in a regime of completely similar sampling and analysis method have not been clarified (Schmidt et al. 2014).

Finally, a wide-ranging research was conducted in Spain to isolate *Salmonella* spp. from cloacal swabs on native reptiles and amphibians, including 35 specimens of snakes of 5 different species, mainly colubrids, out of the 166 animals sampled in total. A total of 27 *Salmonella* were isolated from 19 positive snakes (54.3%), belonging to *S. enterica* subsp*. enterica* (I) and *S. enterica* subsp*. diarizonae* (IIIb) (Briones et al. 2004).

Before the case presented, the Regional Reference Center for Enteropathogens (CREP) serotyped *Salmonella* strains from free-living native snakes (*n* = 18) collected in Central Italy in the period 2010–2012. The serovars found were *S. enterica* subsp*. diarizonae* (IIIb) (*n* = 16, 88.89%) and *S. enterica* subsp*. arizonae* (IIIa) (*n* = 2, 11.11%).

Therefore, the CREP case series shows a substantial affinity with the results obtained in targeted studies conducted in the rest of Europe, with a clear prevalence of *S. enterica* subsp*. diarizonae* (IIIb) in positive European snakes. Furthermore, no other cases of isolation of *S*. Yopougon from food or animals emerged consulting the national database of *Salmonella* isolates of veterinary origin collected by the National Reference Center for Salmonellosis, at the Istituto Zooprofilattico Sperimentale delle Venezie, Legnaro, Italy, during the same period (personal communication). According to data collected for Italy by the ECDC “Surveillance Atlas of Infectious Diseases”, no isolations of *S*. Yopougon from humans have ever been recorded in Europe.

The presence of *S.* Yopougon in such a remote location in the Mediterranean Sea is probably related to the fact that Montecristo Island constitutes an important rest stop for dozens of species of migratory birds coming from Africa (Spina et al. 1993; Montemaggiori et al. 2002).

The movements and migrations of birds represent a remarkable biological phenomenon, but at the same time, they can also determine epizootiological factors (Hubálek 2004). Birds share with humans the peculiarity of traveling among countries and continents within a few hours (Jourdain et al. 2007). It is established that migratory birds are responsible for a large geographical spread of viral, bacterial, and parasitic pathogens (Hubálek 2004; Jourdain et al. 2007; Foti et al. 2009). The efficiency of this geographical diffusion depends on various biotic factors (variety of native vertebrate hosts, invertebrate vectors, resistance of the microorganism in the environment) and abiotic factors (humidity, temperature, etc.), that influence the survival of the etiological agent in the new environment. Migratory birds may be involved in the transport of pathogenic microorganisms through three epidemiological mechanisms: by serving as biological hosts, mechanical carriers, or biological hosts, and mechanical carriers of infected ectoparasites (Hubálek 2004). When birds act as biological hosts, infection by the pathogen can be acute, chronic, latent, or asymptomatic. The latter course, in particular, is often determined by *Salmonella* spp., although few studies have demonstrated this event in migratory birds (Hubálek 2004; Foti et al. 2009). In young specimens of some bird species, the spread of the pathogen is more effective, and the symptoms are more evident than in adults, even in cases of *Salmonella* spp. infections. Moreover, migrations represent an important stress factor for these animals and the immune defenses can be affected, increasing the susceptibility to various pathogens and consequently the duration of their spreading (Hubálek 2004).

At Italian latitudes, from spring to summer, birds arriving from sub-Saharan Africa become numerous, both in number of species and individuals, particularly from March to July, a period that coincides with spring migrations and mating season. This can lead to a greater risk of introduction of pathogens from African continent to Italian latitudes. Most of the sub-Saharan bird species that pass through the Mediterranean Sea in spring are insectivorous passerines, which winter in Africa and mate in Europe (Jourdain et al. 2007). Crossing the Mediterranean may imply the need to fly over large stretches of open sea for hundreds of kilometers and most European passerines potentially possess all the biological characteristics to succeed in this undertaking. During these very energy-consuming journeys, the islands can represent a unique opportunity to stop while crossing the sea.

In 1988, the National Institute for Wild Fauna (Istituto Nazionale per la Fauna Selvatica, INFS) launched the "Small Islands Project" (Progetto Piccole Isole, PPI), which subsequently became an integral part of the “European-African Songbird Migration Network” starting in 1994 to study and characterize the migratory processes of birds across the Mediterranean Sea (Montemaggiori et al. 2002). Montecristo Island is counted among the many islands examined within the PPI: this allowed to draw up a list of migratory species of ornithological interest, which use the island as a stopover before continuing their flight to Northern Europe. The installation of special capture nets (mist-nets) on the island has made it possible to determine the elements (species, number of species, and period of the year) that distinguish migrations in this area of the central Mediterranean (Spina et al. 1993).

Assuming that *S.* Yopougon originated from strains of *S. enterica* native to the country of first isolation (Ivory Coast), it is plausible that this serovar could have reached Montecristo Island through one or more individuals, or one or more species of migratory birds, that regularly stop on the island during their journey. The Western Whip Snake of Montecristo, as previously mentioned, is a reptile that mainly preys on lizards and small passerines. Therefore, it is possible that the snake, tested positive for *S.* Yopougon, may have become infected by preying on an already infected passing African passerine.

In Europe and Italy, the isolation of *Salmonella* spp. from migratory bird species from Africa has been demonstrated on several occasions (Hubálek 2004; Jourdain et al. 2007; Foti et al. 2009, 2011; Mancini et al. 2020).

A recent study on some pathogenic bacteria (*Salmonella* spp., *Campylobacter* spp., and *Yersinia enterocolitica*) was conducted by collecting fecal samples of migratory and sedentary species of birds in the Laghi Lungo e Ripasottile Regional Natural Park of Lazio, from March to December 2012. Out of 92 samples divided into 16 different bird species, 3 were positive for *Salmonella* spp. (3.20%), particularly *S.* Livingstone was isolated in feces from a specimen of blackcap (*Sylvia atricapilla*). Blackcaps are migratory passerines that also come from sub-Saharan Africa and the Ivory Coast (Spina et al. 2022). Their massive presence in Montecristo Island was demonstrated during migration between March and April, within the research context of the PPI (Spina et al. 1993; Svensson et al. 2015; Mancini et al. 2020).

In a previous study conducted in 2006, samples were collected from cloacal swabs and organs of the only species of migratory birds found dead on Ustica Island, off the coast of Sicily. Out of 21 specimens, only 1 *Salmonella bongori* serovar 48z:35 positive bird was identified, once again in a blackcap (Foti et al. 2011). The authors hypothesized correlation between the *S. bongori* strain isolated from the blackcap and two epidemic clusters of human salmonellosis affected children aged between 1 and 2 years with acute enteritis, in Palermo and Messina between the 1980s and 1990s. Molecular investigations demonstrated that the strain responsible for human epidemics shared identical genetic pattern with one from the blackcap of 2006, as well as other strains of the same serovar isolated from two healthy pigeons in southern Italy, also in 2006 (Giammanco et al. 2002; Foti et al. al. 2009). Furthermore, *S. bongori* serovar 48:z35 was recently isolated in Piedmont from a child with acute hemorrhagic enteritis. Based on epidemiological investigation, it is suggested that the patient may have become infected during a trip to Sardinia, although the source of infection could not be traced, and there were no reported contacts with local wildlife. (Bellio et al. 2016).

Very little is still known about the potential commensal bacteria of Italian and European free-living reptiles, especially regarding the pathogenic impact that some of them could have on humans or domestic and wild animals. In this context, a more comprehensive study on the commensal flora of free-living reptiles in Italy is desirable, to assess the risk of zoonosis for humans and transmission to other animal species. Furthermore, research on the microbiota of the fauna of certain natural reserves uninhabited by humans, including Montecristo Island, would be necessary to discover potential pathogens that may have apparently disappeared or never emerged.

### Supplementary Information

Below is the link to the electronic supplementary material.

Supplementary file1 (DOCX 1221 KB)

## Data Availability

Raw reads of the newly found *S.* Yopougon were archived in the GenBank database (NCBI- SRA) under the BioProject PRJNA998331, BioSample SAMN36701244; and in EnteroBase database under the barcode SAL_SB9201AA, name SAL_117300.
