# Supplementary material for: An extremely rare serovar of Salmonella enterica (Yopougon) discovered in a Western Whip Snake (Hierophis viridiflavus) from Montecristo Island, Italy: case report and review
Source: Arch Microbiol. 2024 Jan 3;206(1):49. doi: 10.1007/s00203-023-03772-w (PMC10761451; doi:10.1007/s00203-023-03772-w)
Supplement: Supplementary file 1 — Supplementary file1 (DOCX 1221 KB) [file 203_2023_3772_MOESM1_ESM.docx]

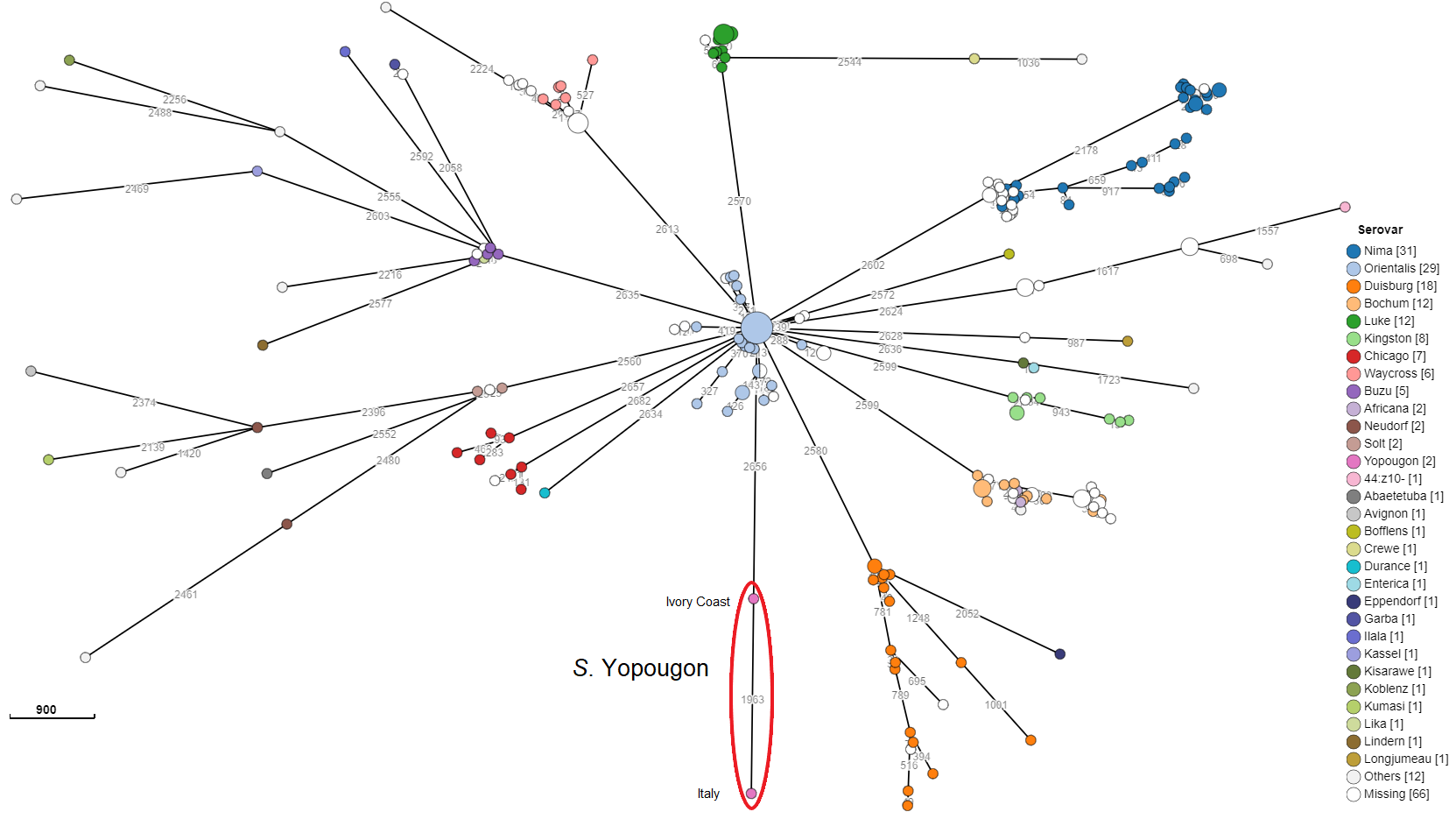
Figure S1. Minimum Spanning Tree of the cgMLST calculated with the most similar strains collected in EnteroBase. Highlighted in red the *S.* Yopougon from the clinical isolate in Ivory Coast (IP 7148/89) and from wild snake in Italy (SAL_117300)
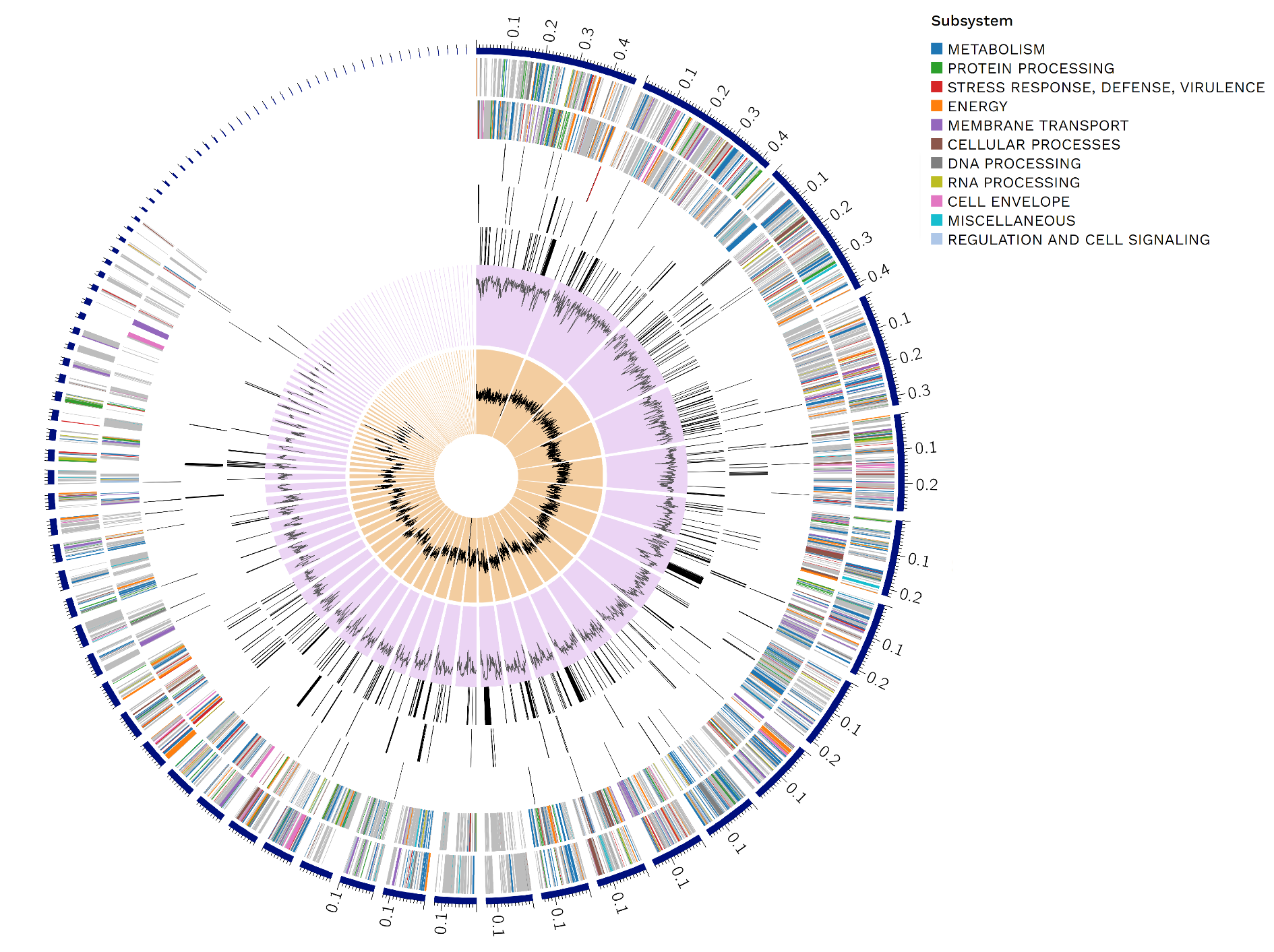


Figure S2. Circular distribution of the genome annotations. From outer to inner rings, the contigs, CDS on the forward strand, CDS on the reverse strand, RNA genes, CDS with homology to known antimicrobial resistance genes, CDS with homology to know virulence factors, GC content and GC skew. The colours of the CDS on the forward and reverse strand indicate the subsystem that these genes belong to.

| Table S1. Core virulence genes found in S. Yopougon isolated from the wild snake | | |
| --- | --- | --- |
| **Virulence factors** | **Related genes** | **Presence** |
|  |  |  |
| ***Adherence*** | |  |
| **Agf** | csgG | + |
|  | csgF | + |
|  | csgE | + |
|  | csgD | + |
|  | csgB | + |
|  | csgA | + |
|  | csgC | + |
| **Bcf** | bcfA | + |
|  | bcfB | + |
|  | bcfC | + |
|  | bcfD | + |
|  | bcfE | + |
|  | bcfF | + |
|  | bcfG | + |
| **Lpf** | lpfE | - |
|  | lpfD | - |
|  | lpfC | - |
|  | lpfB | - |
|  | lpfA | - |
| **MisL** | misL | + |
| **Pef** | pefD | - |
|  | pefC | - |
|  | pefA | - |
|  | pefB | - |
| **Peg** | pegD | - |
|  | pegC | - |
|  | pegB | - |
|  | pegA | - |
| **RatB** | ratB | + |
| **Saf** | safA | + |
|  | safB | + |
|  | safC | + |
|  | safD | + |
| **Sef** | sefA | - |
|  | sefB | - |
|  | sefC | - |
|  | sefD | - |
| **ShdA** | shdA | - |
| **SinH** | sinH | + |
| **Sta** | staG | - |
|  | staF | - |
|  | staE | - |
|  | staD | - |
|  | staC | - |
|  | staB | - |
|  | staA | - |
| **Stb** | stbE | + |
|  | stbD | + |
|  | stbC | + |
|  | stbB | + |
|  | stbA | + |
| **Stc** | stcD | - |
|  | stcC | - |
|  | stcB | - |
|  | stcA | - |
| **Std** | stdC | + |
|  | stdB | + |
|  | stdA | + |
| **Ste** | steA | + |
|  | steB | + |
|  | steC | + |
|  | steD | - |
|  | steE | - |
|  | steF | - |
| **Stf** | stfA | - |
|  | stfC | - |
|  | stfD | - |
|  | stfE | - |
|  | stfF | - |
|  | stfG | - |
| **Stg** | stgA | - |
|  | stgB | - |
|  | stgC | - |
|  | stgD | - |
| **Sth** | sthE | + |
|  | sthD | + |
|  | sthC | + |
|  | sthB | + |
|  | sthA | + |
| **Sti** | stiH | - |
|  | stiC | - |
|  | stiB | - |
|  | stiA | - |
| **Stj** | stjB | - |
|  | stjC | - |
| **Stk** | stkG | - |
|  | stkF | - |
|  | stkE | - |
|  | stkD | - |
|  | stkC | - |
|  | stkB | - |
|  | stkA | - |
| **Tcf** | tcfA | - |
|  | tcfB | - |
|  | tcfC | - |
|  | tcfD | - |
| **Type 1 fimbriae** | fimA | + |
|  | fimI | + |
|  | fimC | + |
|  | fimD | + |
|  | fimH | + |
|  | fimF | + |
|  | fimZ | + |
|  | fimY | + |
|  | fimW | + |
| ***Effector delivery system*** | |  |
| **SCI (Salmonella centrisome island)** | tssA | + |
|  | TssB | + |
|  | TssC | + |
|  | TssD/Hep | - |
|  | tssE | - |
|  | tssF | + |
|  | tssG | + |
|  | TssK | + |
|  | TssH/clpV | - |
|  | PAAR | - |
|  | TssI/vgrG | - |
|  | TssJ | + |
|  | tssM | + |
|  | tssL | - |
| **TTSS (SPI-1 encode)** | sprB | + |
|  | hilC | + |
|  | orgC | + |
|  | orgB/SctL | + |
|  | orgA/sctK | + |
|  | prgK | + |
|  | prgJ | + |
|  | prgI | + |
|  | prgH | + |
|  | hilD | + |
|  | hilA | + |
|  | iagB | + |
|  | sicP | + |
|  | iacP | + |
|  | sipD | + |
|  | sicA | + |
|  | spaS | + |
|  | spaR | + |
|  | spaQ | + |
|  | spaP | + |
|  | spaO/sctQ | + |
|  | invJ | + |
|  | invI | + |
|  | invC/sctN | + |
|  | invB | + |
|  | invA | + |
|  | invE | + |
|  | invG | + |
|  | invF | + |
|  | invH | + |
| **TTSS (SPI-2 encode)** | ssrB | + |
|  | ssrA | + |
|  | ssaC | + |
|  | ssaD | + |
|  | ssaE | + |
|  | sseA | + |
|  | sseB | + |
|  | sscA | + |
|  | sseC | + |
|  | sseD | + |
|  | sseE | + |
|  | sscB | + |
|  | ssaG | + |
|  | ssaH | + |
|  | ssaI | + |
|  | ssaJ | + |
|  | ssaK | + |
|  | ssaL | + |
|  | ssaM | + |
|  | ssaV | + |
|  | ssaN | + |
|  | ssaO | + |
|  | ssaP | + |
|  | ssaQ | + |
|  | ssaR | + |
|  | ssaS | + |
|  | ssaT | + |
|  | ssaU | + |
| **TTSS effectors secreted via both systems** | slrP | + |
| **TTSS-1 secreted effectors** | sptP | + |
|  | sipA/sspA | + |
|  | sipC/sspC | + |
|  | sipB/sspB | + |
|  | sopA | + |
|  | sopB/sigD | + |
|  | sopD | + |
|  | sopE | - |
|  | sopE2 | + |
|  | avrA | + |
| **TTSS-2 secreted effectors** | spiC/ssaB | + |
|  | sseF | + |
|  | sseG | + |
|  | sseI/srfH | - |
|  | sseJ | + |
|  | sseL | + |
|  | sseK2 | + |
|  | sifA | + |
|  | sifB | + |
|  | pipB | + |
|  | pipB2 | + |
|  | sopD2 | + |
|  | gogB | - |
|  | sspH2 | - |
|  | sseK1 | + |
|  | spvD | - |
|  | spvC | - |
| ***Exotoxin*** | |  |
| **SpvB** | spvB | - |
| **Typhoid toxin** | cdtB | + |
|  | pltA | + |
|  | pltB | + |
| ***Immune modulation*** | |  |
| **Rck** | rck | - |
| **Vi antigen** | vexE | - |
|  | vexD | - |
|  | vexC | - |
|  | vexB | - |
|  | vexA | - |
|  | tviE | - |
|  | tviD | - |
|  | tviC | - |
|  | tviB | - |
|  | tviA | - |
| ***Nutritional/Metabolic factor*** | |  |
| **MgtBC** | mgtB | + |
|  | mgtC | + |
| ***Stress survival*** | |  |
| **SodCI** | sodCI | - |
| ***Antimicrobial activity/Competitive advantage*** | |  |
| **Mig-14** | mig-14 | + |
| **Mig-5** | mig-5 | - |
| ***Regulation*** | |  |
| **PhoPQ** | phoQ | + |
|  | phoP | + |
